# Effects of Zymosan on Short-Chain Fatty Acid and Gas Production in *in vitro* Fermentation Models of the Human Intestinal Microbiota

**DOI:** 10.3389/fnut.2022.921137

**Published:** 2022-07-04

**Authors:** Xionge Pi, Zaichun Yu, Xiaoxia Yang, Zhi Du, Wei Liu

**Affiliations:** ^1^Institute of Plant Protection and Microbiology, Zhejiang Academy of Agricultural Sciences, Hangzhou, China; ^2^College of Bioengineering, Zhejiang University of Technology, Hangzhou, China; ^3^Department of Pharmacy, The Children's Hospital, Zhejiang University School of Medicine, National Clinical Research Center for Child Health, Hangzhou, China

**Keywords:** zymosan, intestinal microbiota, *in vitro* fermentation, short-chain fatty acids, gas

## Abstract

In this study, the effects of zymosan (HG, hydrolyzed glucan) on the structure and metabolism of fecal microbiota in Chinese healthy people was investigated by an *in vitro* simulated intestinal microecology fermentation model. We found that HG significantly regulated fecal microbiota composition, including the increase of *Bifidobacterium, Faecalibacterium, Prevotella* and the decrease of *Escherichia-Shigella*. Moreover, HG significantly increased the total production of short chain fatty acids (SCFAs) and gases, in which the production of Acetic acid, Propionic acid, CO_2_, and H_2_ significantly increased while the production of Isovaleric acid and NH_3_ significantly decreased. Additionally, the supplement of HG showed certain differences in the regulation of microbiota from four groups. HG significantly increased the relative abundance of *Bifidobacterium* and significantly decreased the relative abundance of *Escherichia-Shigella* excluding the older men group. Meanwhile, and the relative abundance of *Lactobacillus* was significantly increased in young populations. And the relative abundance of *Bacteroides* was significantly decreased only in the young women. Furthermore, HG significantly increased H_2_ concentration only in older men. These findings suggest that HG, as a new generation of prebiotics, could regulate the structure of fecal microbiota and its metabolites in a better direction, but when HG participates in precision nutrition formula, it may be necessary to consider the differences in the utilization of different populations.

## Introduction

Recently, with deepening understanding of intestinal microbiota, the relationship between intestinal microbiota and human health has attracted attention. The human intestinal microbiota is composed of nearly one trillion microorganisms, whose diversity and composition are often closely correlated with human health (1). Different dietary habits, age and gender have been shown to lead to different compositions of the intestinal microbiota (2). Some yeasts, such as *Saccharomyces boulardii* and *Saccharomyces baumannii* play an important role in human intestines and human health (3, 4). For instance, yeast glucans from yeast cell walls were associated with many prebiotic roles, including anti-tumor, constipation relieving, cholesterol lowering, and gastrointestinal regulation. As an immune enhancer, zymosan is widely used as a feed additive in the field of aquaculture and animal husbandry (5, 6). Besides, it has also been used as a thickener and emulsifier in industries (7).

Zymosan significantly reduces the number of pathogenic bacteria, such as *Salmonella* and *Escherichia coli* (8), promoted the proliferation of beneficial bacteria, and increased the contents of total short-chain fatty acids (SCFAs) in pig intestines (9), thereby exerting beneficial effects on the body. Moreover, zymosan effectively improved the intestinal permeability and structural integrity of tight junctions in mice with ulcerative colitis (10). The importance of zymosan on animal intestinal microbiota may contribute to human intestinal health. However, the direct effects of zymosan on human intestinal microbiota have not been conclusively determined.

We evaluated the effects of zymosan on human intestinal microbiota and their metabolites *via* an *in vitro* batch fermentation model. Our findings provide a theoretical basis for the use of zymosan as a functional food.

## Materials and Methods

### Materials and Reagents

Zymosan (HG, hydrolyzed glucan), a glucan with good water solubility, was obtained by moderate enzymatic hydrolysis of yeast β-glucan. It was provided by Angel Yeast Co. Ltd. It was obtained as: *Saccharomyces cerevisiae* was cultured, utolyzed, enzymatically hydrolyzed and centrifuged to obtain yeast cell walls and contents. The cell wall was diluted to a concentration of 10% after a certain amount of sodium hydroxide was added, heat treated for 4–6 h, centrifuged, re-dispersed for washing, and spray-dried. Zymosan was obtained by collecting them, and the content with molecular weight in the range of 200–300 kDa is about 95% with good water solubility. Tryptone, yeast extract, L-cysteine, NaCl, KH_2_PO_4_, K_2_HPO_4_, heme, MgSO_4_, CaCl_2_, and crotonic acid were purchased from Sigma Company, USA.

### Sampling Population and Collection of Fecal Samples

Forty-one volunteers were recruited from local healthy people in Hangzhou (who had no intestinal diseases and had not been administered with antibiotics, prebiotics, and probiotics, et al. in the last 4 weeks). This study was approved by the Ethical Committee of the Hangzhou Center for Disease Control and Prevention (No. 202047). Those aged 20–30 formed the young group, while those aged 40–60 formed the older group. There were 10 people in the young women group (YW), older men group (OM), and older women group (OW). There were 11 people in the young men group (YM). The baseline characteristics of the volunteers included in the study are shown in Table 1. They were provided with sterile stool sampling boxes and asked to quickly pick up no <3 g intermediate stool with less food residues and less oxygen exposure during defecation, mark the name, age and sampling date of the donor. The collected samples were stored at 4°C and assayed within 4 h.

**Table 1:** Baseline characteristics of the selected volunteers.

**Group**	**Number**	**Age range/year**	**Gender**	**Gastrointestinal disease**	**Taking antibiotics and other medicines**
Young men	11	20–30	Male	None	None
Older men	10	40–60	Male	None	None
Young women	10	20–30	Female	None	None
Older women	10	40–60	Female	None	None

### Pre-treatment of Fecal Samples

We separately weighed 0.2 g of fresh fecal samples from fecal sampling boxes into three 1.5 mL sterile centrifuge tubes and stored them in a −80°C freezer as raw fecal samples. Then, 0.8 g of the fecal samples were weighed into 10 mL sterile centrifuge tubes, 8 mL of sterile PBS buffer solution was added, and the interfaces sealed using a tape. Finally, fecal and buffer solutions were mixed in the shaker, and the supernatant collected by filtration to obtain a 10% fecal suspension inoculum.

### Growth Conditions of Fecal Microbiota

In this study, *in vitro* fecal fermentation was performed according to the method of Zhao et al. (11). The blank control (Ctrl) medium contained: 10 g/L tryptone, 2.5 g/L yeast extract, 1 g/L L-cysteine, 2 mL/L heme solution, 0.9 g/L NaCl, 0.09 g/L CaCl_2_·6H_2_O, 0.45 g/L KH_2_PO_4_, 0.45 g/L K_2_HPO_4_, and 0.09 g/L MgSO_4_·7H_2_O. Immediately after dissolving and boiling, nitrogen was filled to keep the liquid surface of the medium free of oxygen. Then, 4.5 mL culture medium was dispensed into vials using a peristaltic pump. Finally, the vial was sealed with a cap and sterilized by high pressure steaming. HG medium is to add HG to Ctrl medium at a ratio of 0.8 g/100 mL.

### *In vitro* Fermentation of Fecal Microbiota

In the anaerobic bench, 500 μL of the fresh fecal suspension inoculum was inoculated into 4.5 mL HG medium and Ctrl medium, respectively, using sterile disposable syringes. Then, they were incubated at 37°C for 24 h and stored in a refrigerator at 4°C for use in fermentation. The same group of experiments was conducted three times in parallel.

### DNA Extraction and 16S rRNA Sequencing of Fecal and Fermentation Samples and Bioinformatic Analysis

Microbial community genomic DNA was extracted from fecal and fermentation samples using FastDNA® Spin Kit for Soil (MP Biomedicals, U.S.), according to the manufacturer's instructions. On a thermocycler PCR system (GeneAmp 9700, ABI, San Diego, CA, USA), the V3–V4 hypervariable regions of bacterial 16S rRNA gene were amplified with primers 341F (5′-CCTAYGGGRBGCASCAG-3′) and 806R (5′-GGACTACHVGGGTWTCTAAT-3′). The PCR conditions were: pre-denaturation for 3 min, 27 cycles at 95°C for 30 s, annealing at 55°C for 30 s, elongation at 72°C for 45 s, and 10 min extension at 72°C. Purified amplicons were pooled in equimolar and paired-end sequenced on a NovaSeq PE250 platform (Illumina, San Diego, USA) according to standard protocols by Majorbio Bio-Pharm Technology Co. Ltd. (Shanghai, China).

Amplicon sequence variants (ASVs) were obtained by denoising the optimized sequences after quality control splicing using the DADA2 (12) plugin in the Qiime2 (version 2020.2) (13) process. Taxonomic assignment of ASVs was performed using the Naive bayes consensus taxonomy classifier implemented in Qiime2 and the SILVA 16S rRNA database (v138). All consensus sequence data of raw fecal samples and fermentation samples were submitted to the National Center for Biotechnology Information Short Read Archive under accession no. PRJNA774123.

### Determination of Fermented Short-Chain Fatty Acids

A 2.5 g of metaphosphoric acid was dissolved in 100 mL deionized water to prepare 100 mL metaphosphoric acid solution that was supplemented with 0.6464 g crotonic acid to prepare a crotonic acid/metaphosphoric acid solution. The fermentation broth (500 μL) and crotonic acid/metaphosphoric acid solution (100 μL) were evenly mixed and stored in a −40°C refrigerator for acidification for 24 h. After acidification, samples were centrifuged for 3 min to separate the supernatants from the precipitate at a speed of 13,000 r/min at 4°C, and filtered *via* a 0.22 μm hydrophilic micron membrane. Then, 150 μL of the filtered solution was aspirated into a sample vial.

The gas chromatograph was prepared, loaded with the sample after which the aging procedure was performed. Column temperature heating conditions were: column temperature: 80°C for 1 min, 10°C/min, increased to 190°Cand maintained for 0.50 min; then increased to 240°C at a rate of 40°C/min and maintained for 5 min; FID detector: 240°C; gasification chamber: 240°C; carrier gas: nitrogen flow rate 20 mL/min, hydrogen flow rate 40 mL/min, air flow rate 400 mL/min. The obtained data were recorded.

### Fermentation Gas Analysis

After fermentation, the vial was restored to room temperature after which the gas was automatically analyzed using a gas analyzer to determine gas composition and concentration (14).

### Data Analysis

The microflora data were analyzed on the online platform of Majorbio Cloud Platform (www.majorbio.com). The alpha diversity was determined by the Wilcoxon rank-sum test between the two groups. And the beta diversity (PCoA and NMDS graphs) was based on the ASV table and bray-curtis distance algorithm to analyze the structural changes of the microbial community at the genus level. Statistical comparisons of taxa abundances at the phylum and genus levels for each group were performed and displayed as Veen and Bar plots. The correlation Heatmap used the correlation coefficient Spearman to evaluate the correlation of each bacterial genera in the fermentation group with SCFAs and gases. KEGG functional prediction analysis was performed by PICRUSt2. GraphPad Prism 8.0.1 was used for data analysis and mapping of gases, SCFAs, four bacterial genera and metabolic functions. SPSS 23.0 was used for data and statistical significance analysis. The Shapiro-Wilk normality test was used to determine whether the data fit a normal distribution. For normally distributed data, paired *t*-test was performed between two groups, and one-way ANOVA followed by Tukey's multiple comparison test was performed between multiple groups (four populations). The paired Wilcoxon rank test was performed between two groups and the Kruskal-Wallis test was performed between multiple groups when data did not conform to a normal distribution. All results are presented as mean ± SEM (41 independent experiments × 3 parallel experiments). Significant differences were defined as *p* ≤ 0.05.

## Results

### Composition Analysis of Fecal Microbiota Before and After Fermentation

**Figure 1:**
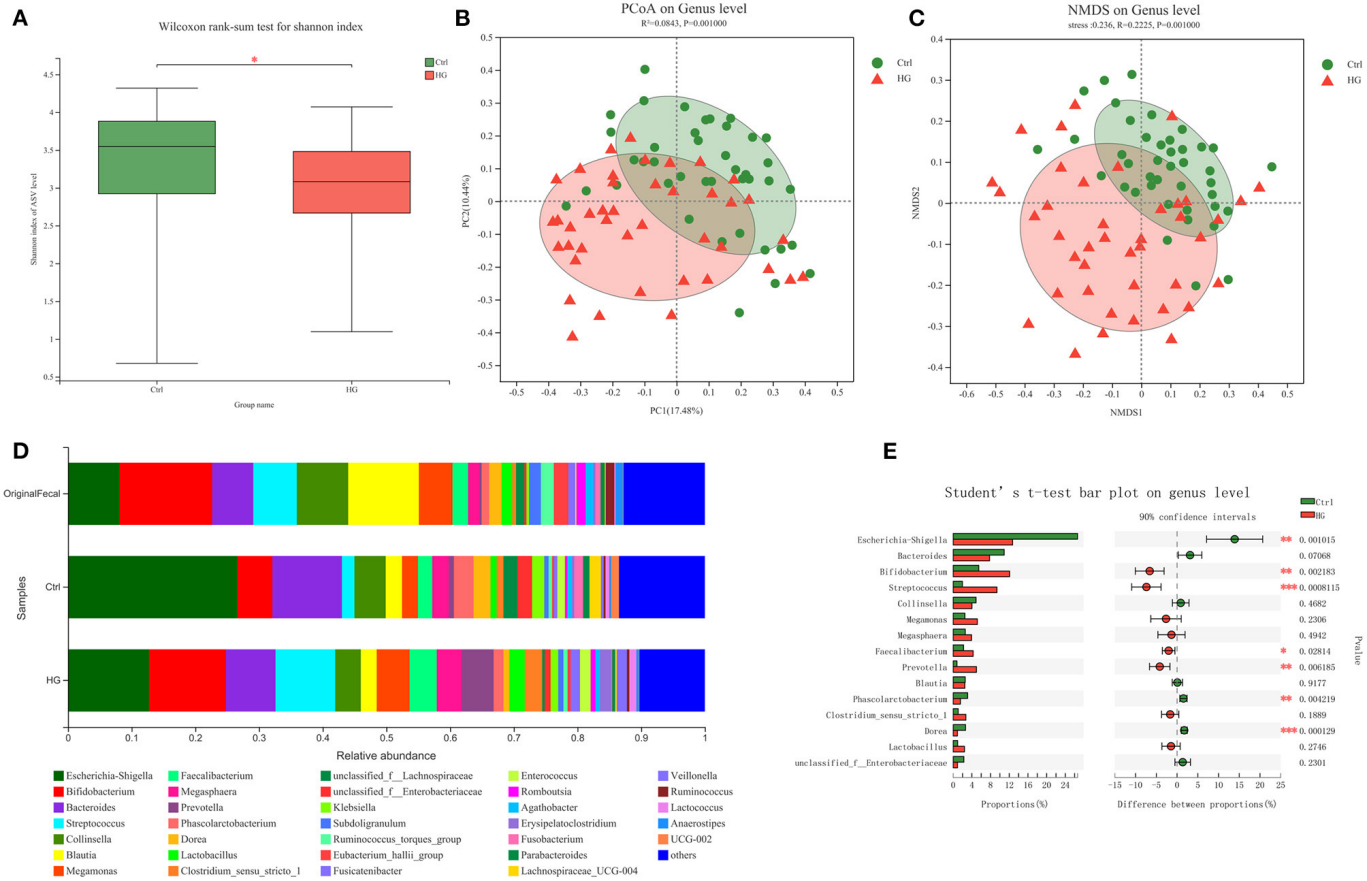
Composition of original fecal microbiota and the microbiota after fermentation. **(A)** Alpha-diversity analysis and **(B)** Beta-diversity PCoA analysis and **(C)** NMDS analysis of Ctrl group and HG group; **(D)** Microbiota composition of original fecal samples and fermentation groups; **(E)** Abundance comparison at genus level between Ctrl group and HG group. Data are means ± SEM (41 independent experiments × 3 replication experiments). Statistical significance thresholds were: *0.01 < *p* ≤ 0.05; **0.001 < *p* ≤ 0.01; ***p ≤ 0.001.

The 16S rRNA sequencing technology was used to analyze variations of fecal microbiota in different populations before and after fermentation. After fermentation, the alpha-diversity of the Ctrl group and the HG group was shown in Figure 1A. The Shannon index of the HG group was significantly decreased (*P* = 0.032), indicating that the community diversity of the HG group was significantly lower than that of the Ctrl group. In addition, PCoA analysis and NMDS analysis showed that there was a significant difference in the genus-level bacterial community structure between the Ctrl group and the HG group after fermentation (*P* = 0.001, Figures 1B,C). As shown in Figure 1D, at the genus level, *Bifidobacterium* had the highest abundance in original fecal samples, followed by *Blautia, Collinsella*, and *Escherichia-Shigella*. The abundance of these bacteria also varied in the original fecal samples of the four populations. However, the 15 bacterial genera with the highest relative abundance did not show statistical differences among the four populations (Supplementary Figure 2). After 24 h of fermentation, compared to the Ctrl group, the relative abundances of *Bifidobacterium* (*P* = 0.002), *Streptococcus* (*P* < 0.001), *Faecalibacterium* (*P* = 0.028) and *Prevotella* (*P* = 0.006) were significantly increased in the HG group. While the relative abundance of *Escherichia-Shigella* (*P* = 0.001), *Phascolarctobacterium* (*P* = 0.004) and *Dorea* (*P* < 0.001) in the HG group were significantly lower than in the Ctrl group (Figure 1E).

**Figure 2:**
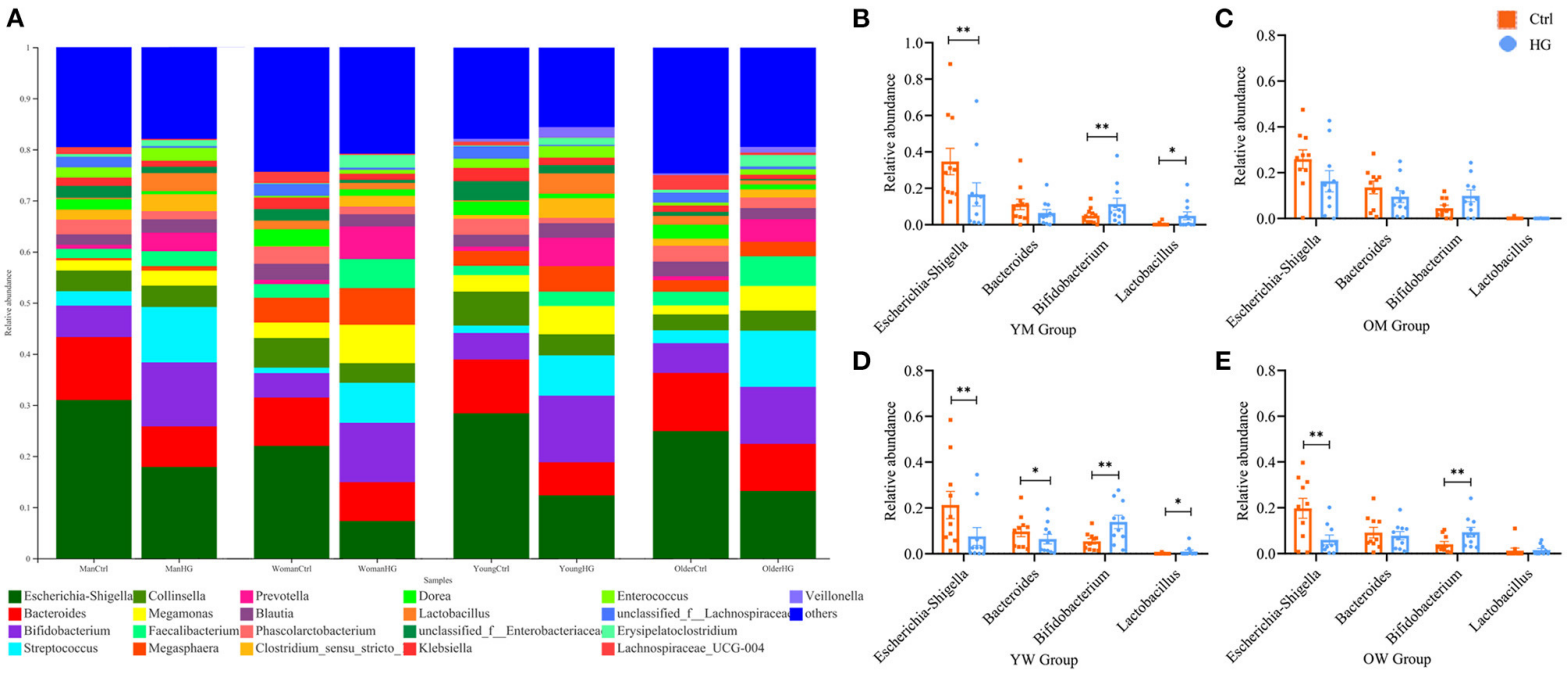
Bacterial composition of four populations in two media after fermentation. **(A)** Genus-level microbiota composition for all groups; the relative abundance ratios of the four bacteria in **(B)** YM, **(C)** OM, **(D)** YW, and **(E)** OW after fermentation. Data are means ± SEM (41 independent experiments × 3 replication experiments). Statistical significance thresholds were: *0.01 < *p* ≤ 0.05; **0.001 < *p* ≤ 0.01.

We further analyzed the effect of gender and age on the fermentation results (Figure 2). The results showed that the trend of relative abundance of mainly bacteria at genus level was independent of gender and age. In the HG group, the relative abundance of *Escherichia-Shigella* decreased and the relative abundance of *Bifidobacterium* increased in all populations. The three bacterial genera with the highest abundance after fermentation are *Escherichia-Shigella, Bacteroides, Bifidobacterium*, and *Lactobacillus* is a probiotic that is often studied. We performed statistical tests on the relative abundance of these four bacteria in the two media. The results showed that the relative abundance of *Bifidobacterium* was significantly increased in the young men (*P* = 0.004), the young women (*P* = 0.002) and the older women population (*P* = 0.009). While the relative abundance of *Escherichia-Shigella* was significantly decreased in the young men (*P* = 0.003), the young women (*P* = 0.005) and the older women population (*P* = 0.009). Additionally, in the HG group, the relative abundance of *Lactobacillus* was significantly increased in young men (*P* = 0.025) and young women population (*P* = 0.040). And the relative abundance of *Bacteroides* was significantly decreased only in the young women population (*P* = 0.04). For the older men population, the effects of HG on these four bacteria did not show statistical significance. Therefore, fecal microbiota in different populations varied differentially in response to HG and were susceptible to gender and age.

### SCFAs Production During Fecal Microbiota Fermentation

**Figure 3:**
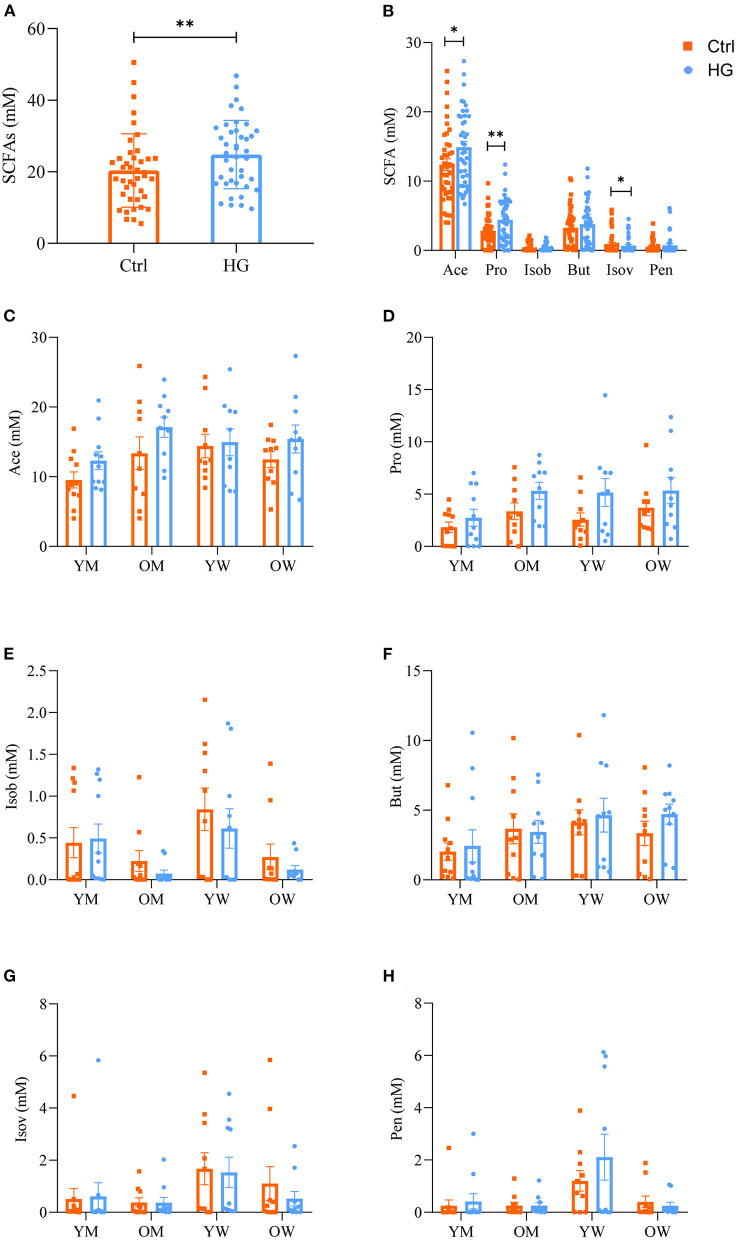
Effects of HG on the levels of SCFAs produced by fecal microbiota in different populations in the *in vitro* fermentation model. The levels of total acids **(A)** and six SCFAs **(B)** produced by the fecal microbiota from all populations, and the levels of acetic acid **(C)**, propionic acid **(D)**, isobutyric acid **(E)**, butyric acid **(F)**, isovaleric acid **(G)**, and pentanoic acid **(H)** produced by the fecal microbiota in the four populations. Data are means ± SEM (41 independent experiments × 3 replication experiments). Statistical significance thresholds were: *0.01 < *p* ≤ 0.05; **0.001 < *p* ≤ 0.01.

Fecal microbiota of different populations generated a lot of SCFAs after 24 h fermentation. As shown in Figure 3, the levels of six SCFAs in fermentation broth, including acetic acid (Ace), propionic acid (Pro), isobutyric acid (Isob), butyric acid (But), isovaleric acid (Isov), and pentanoic acid (Pen), were determined. Compared with the Ctrl group, the levels of total SCFAs were significantly increased in the HG group (Figure 3A). The levels of Ace were higher than those of the other SCFAs, followed by Pro and But (Figure 3B). Levels of total SCFAs (*P* = 0.006), Ace (*P* = 0.023), and Pro (*P* = 0.003) in the HG medium were significantly increased. While the levels of Isov (*P* = 0.034) was significantly decreased.

Collectively, these observations indicated that the varying trends of SCFAs in different populations are not identical after HG intervention based on gender and age. Compared with the Ctrl group, HG relatively increased the levels of Ace and Pro in different populations (Figures 3C,D), and relatively reduced the levels of Isob and Isov in some women (Figures 3E,G). The levels of But were relatively higher in young women and old women in the HG group (Figure 3F). In addition, HG only relatively increased the Pen levels in some young women and men (Figure 3H).

### Gas Production During Fecal Microbiota Fermentation

**Figure 4:**
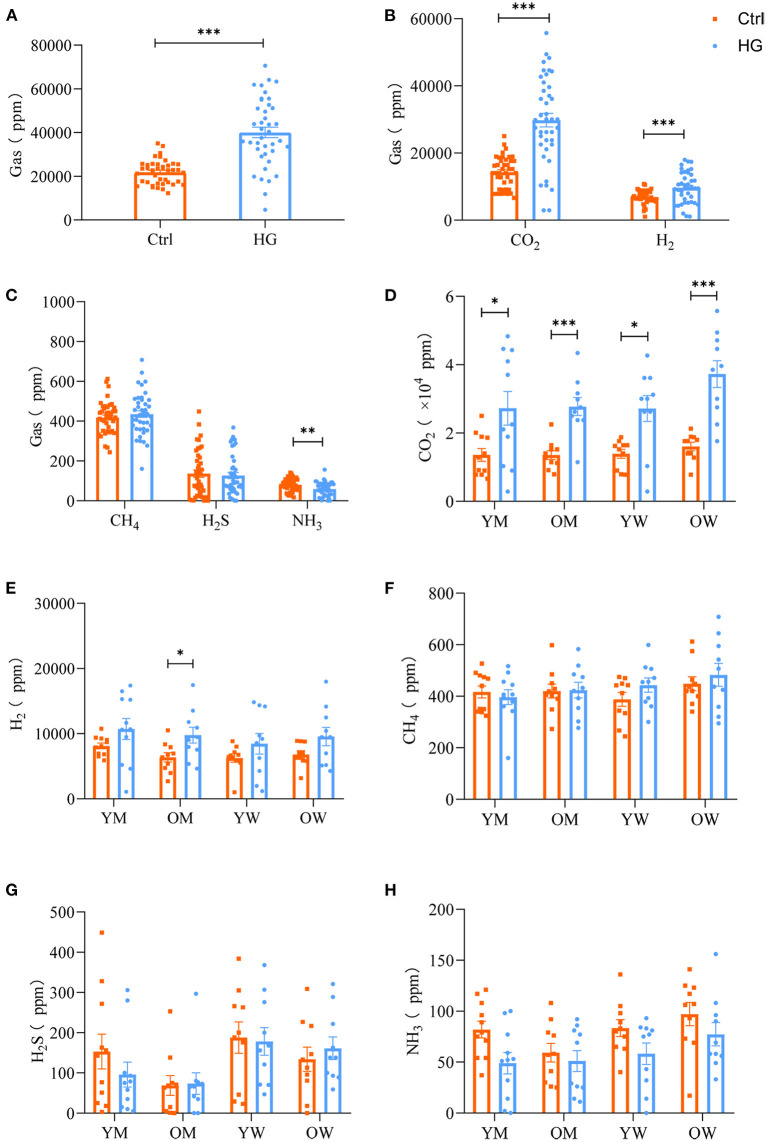
Effects of HG on the concentration of gas produced by fecal microbiota in different populations in the *in vitro* fermentation model. The concentration of total gases **(A)** and five gases **(B,C)** produced by the fecal microbiota from all populations, and the concentration of CO_2_ **(D)**, H_2_ **(E)**, CH_4_ **(F)**, H_2_S **(G)**, and NH_3_ **(H)** produced by fecal microbiota in the four populations. Data are means ± SEM (41 independent experiments × 3 replication experiments). Statistical significance thresholds were: *0.01 < *p* ≤ 0.05; **0.001 < *p* ≤ 0.01; ***p ≤ 0.001.

The composition and content of gas produced during fecal microbiota fermentation are shown in Figure 4. After 24 h of fermentation by fecal microbiota, the HG medium of the experimental group and Ctrl medium of the control group had large amounts of CO_2_, H_2_ and small amounts of H_2_S, CH_4_, and NH_3_. And the concentrations of gas in the HG group was significantly higher than that in the Ctrl group (*P* < 0.001, Figure 4A). CO_2_, as the most abundant gas produced by fecal microbiota, was significantly higher in the HG group, relative to the Ctrl group (*P* < 0.001). Additionally, the concentrations of H_2_ in the HG group were significantly increased (*P* < 0.001, Figure 4B), while the concentrations of NH_3_ in the HG group were significantly lower than those of the Ctrl group (*P* = 0.002). Differences in concentrations of CH_4_ and H_2_S were insignificant between the Ctrl and HG group (Figure 4C).

There were no statistically significant differences among the four populations for the five gases in the Ctrl group or HG group. And the concentrations of CH_4_, H_2_S, and NH_3_ did not exhibit differences in each population group (Figures 4F–H). However, compared with the Ctrl group, the supplement of HG significantly increased the concentration of CO_2_ in different populations. Of note, fecal microbiota in the older women group produced relatively higher concentrations of CO_2_ than the other groups. In the HG group, the concentrations of CO_2_ in young men (*P* = 0.028), older men (*P* < 0.001), young women (*P* = 0.010), and older women (*P* < 0.001) was significantly higher than those in the Ctrl group (Figure 4D). The concentrations of H_2_ for older men in the HG group were also significantly increased (*P* = 0.040, Figure 4E). In addition, the concentrations of NH_3_ were relatively high in the women group relative to the men group (Figure 4H).

### Correlations Between Fecal Microbiota and Metabolites During Fermentation

**Figure 5:**
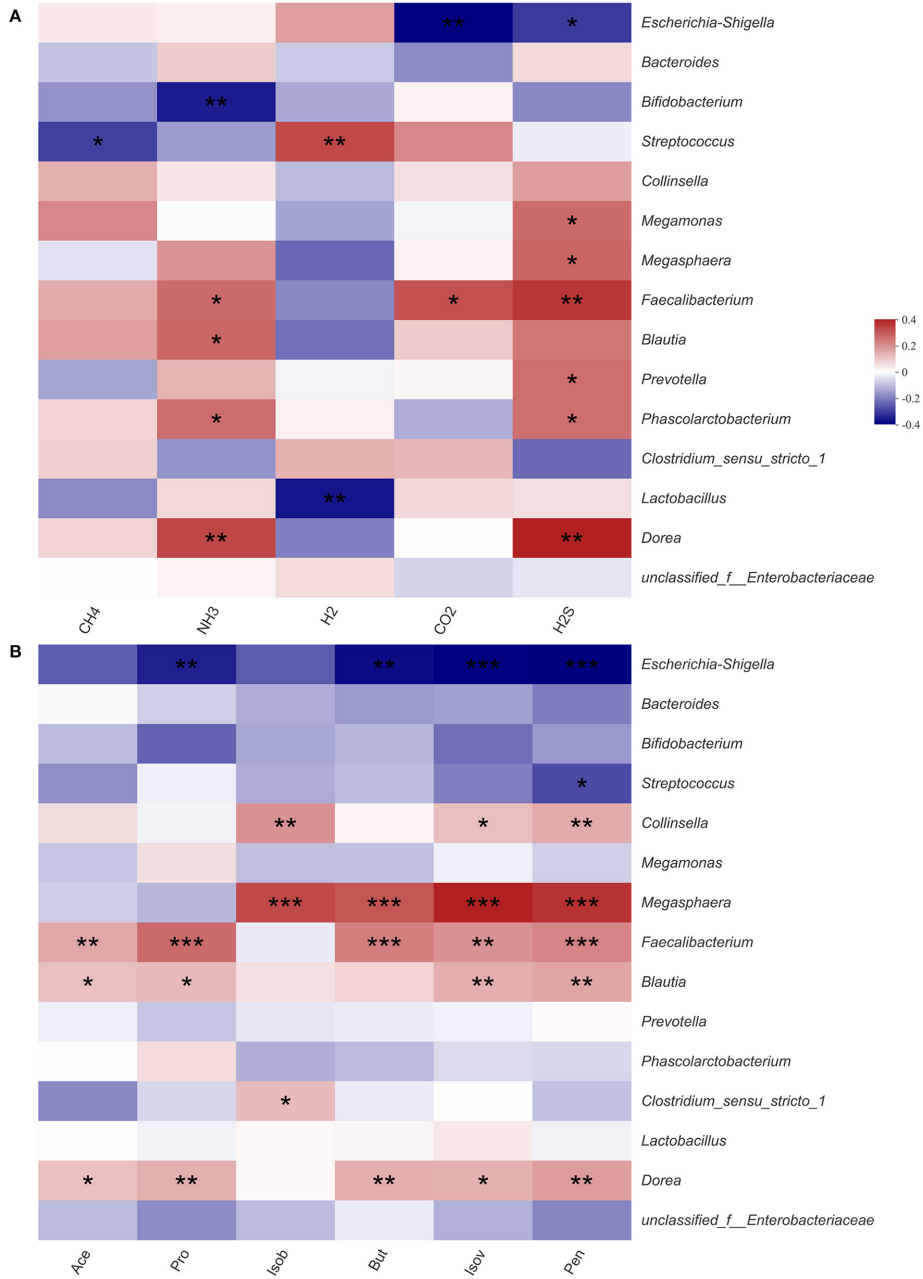
Correlation between fecal microbiota and gas **(A)** and short-chain fatty acid **(B)** in fermentation samples. Data are means ± SEM (41 independent experiments × 3 replication experiments). Statistical significance thresholds were: *0.01 < *p* ≤ 0.05; **0.001 < *p* ≤ 0.01; ***p ≤ 0.001.

Previous experimental results showed that HG could significantly affect the microbiota structure and the production of SCFAs and gases. Therefore, we used heatmaps and Spearman correlation coefficients to analyze correlations between gases, SCFAs and fecal microbiota (Figure 5). Among the top 15 fecal microbiota in genus-level abundance, all gases and SCFAs were jointly correlated various bacteria, except for methane, which was only significantly negatively correlated with *Streptococcus*. The concentrations of H_2_S were found to be correlated with the most bacteria. With regards to individual bacteria, there was no bacterial genus that had marked effects on the production of all gases or SCFAs, however, *Faecalibacterium* had the most extensive influence and could significantly affect the production of NH_3_, CO_2_, H_2_S, Ace, Pro, But, Pen, and Isov. *Escherichia-Shigella*, which accounted for the highest relative abundance, had a significant negative correlation with the concentrations of CO_2_, H_2_S, Pro, But, Pen, and Isov. As two probiotics, *Bifidobacterium* was only significantly negatively correlated with NH_3_, while *Lactobacillus* was only significantly negatively correlated with H_2_.

### Functional Predictive Analysis of Fecal Microbiota During Fermentation

**Figure 6:**
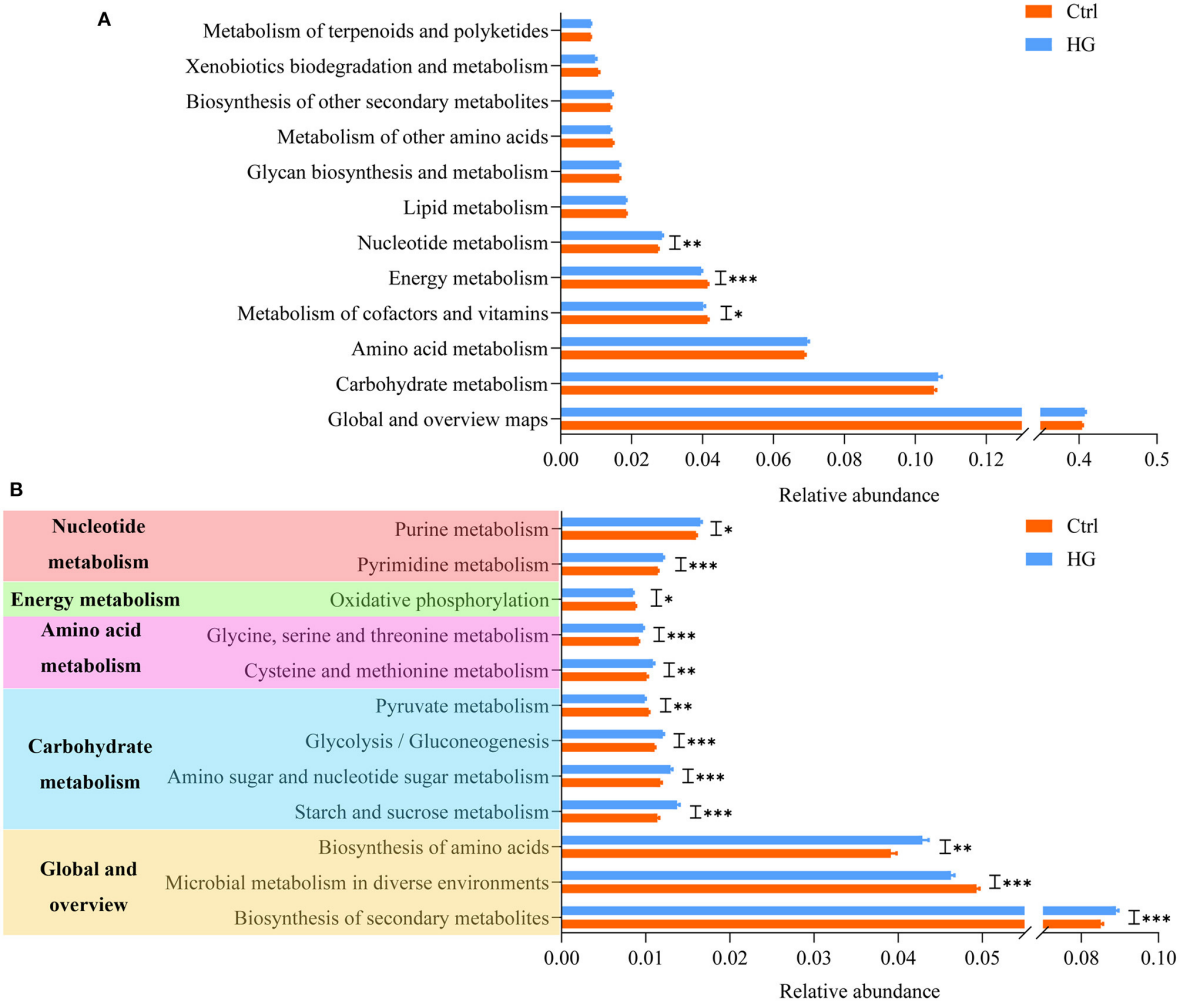
Variation in bacteria function profiles during anaerobic fermentation analyzed by PICRUSt2: **(A)** metabolic pathways abundance on KEGG categories (level 2); **(B)** metabolic pathways abundance on KEGG categories (level 3). Data are means ± SEM (41 independent experiments × 3 replication experiments). Statistical significance thresholds were: *0.01 < *p* ≤ 0.05; **0.001 < *p* ≤ 0.01; ***p ≤ 0.001.

On the basis of 16S rRNA sequencing data, we used PICRUSt2 to predict the metabolic functional of the fecal microbiota, and the results are shown in Figure 6. At level 2, nucleotide metabolism (*p* = 0.003) was significantly improved in the HG group compared with the Ctrl group. While energy metabolism (*P* < 0.001) and metabolism of cofactors and vitamins (*p* = 0.024) were significantly lower in the HG group. There were no significant differences in other metabolic functions. In order to ensure the accuracy, we only analyzed the metabolic functions of 15 bacterial groups whose relative abundance accounted for more than 1% at the level 3. Twelve of these metabolic functions were significantly different between the HG and Ctrl groups (Figure 6B). Biosynthesis of secondary metabolites (*P* < 0.001), biosynthesis of amino acids (*P* = 0.002), starch and sucrose metabolism (*P* < 0.001), amino sugar and nucleotide sugar metabolism (*P* < 0.001), glycolysis/gluconeogenesis (*P* < 0.001), cysteine and methionine metabolism (*p* = 0.006), glycine, serine, and threonine metabolism (*P* < 0.001), pyrimidine metabolism (*P* < 0.001), and purine metabolism (*p* = 0.020) were all significantly elevated in the HG group. However, the relative abundances of genes related to microbial metabolism in diverse environments (*P* < 0.001), pyruvate metabolism (*p* = 0.001), and oxidative phosphorylation (*p* = 0.017) were significantly reduced in the HG group.

## Discussion

There are nearly one trillion microorganisms in the human intestines, and the diversity and composition of these microbiota are often closely correlated with human health (1). *In vitro* simulated fermentation models of the intestinal have been often used to analyze the effects of functional substances on the intestinal microbiota (15–18). We analyzed the effects of HG on fecal microbiota and metabolite compositions in different populations of varying age and genders using an *in vitro* fermentation model.

There were variations in microbiota structure before and after fermentation. Before fermentation, *Bifidobacterium* accounted for the highest abundance of fecal microbiota, which is also the main intestinal commensal bacteria colonizing human infants since birth (19). The second genus with the highest relative abundance was *Blautia*, an anaerobic bacterium with probiotic characteristics (20). However, in the Ctrl group after fermentation, *Escherichia-Shigella* and *Bacteroides* had the highest abundances. The abundance of these two bacteria in the intestines of colorectal cancer patients exhibited an increasing trend (21, 22). And *Escherichia-Shigella* are harmful to the intestinal mucosa (23). After completion of fermentation, compared to the Ctrl group, HG significantly decreased the abundance of *Escherichia-Shigella* and relatively decreased the abundance of *Bacteroides*. Besides, the relative abundance of *Bifidobacterium*, an important probiotic for maintaining intestinal homeostasis (24), was significantly increased. Some species of *Streptococcus* such as *S. thermophiles* and *S. salivarius* are used as probiotics (25). *Faecalibacterium* can serve as a marker for healthy intestinal and can be associated with anti-inflammatory properties (26, 27). And the relative abundances of *Streptococcus* and *Faecalibacterium* were also significantly increased in the HG group. In addition, as important producers of acetate and propionate, the relative abundance of *Prevotella* (28) significantly increased. These results indicate that HG enriched and increased the abundance of beneficial bacteria such as *Bifidobacterium* and *Prevotella* in the intestines, reduced the abundance of harmful bacteria such as *Escherichia-Shigella* and *Bacteroides*, and finally improved the imbalance of fecal microbiota.

The abundance of fecal microbiota from different groups exhibited certain differences, which were established to be significant after fermentation (Supplementary Figure 1), indicating that the regulation ability of HG on fecal microbiota is affected by gender and age. For instance, it may be concluded from Figure 2 that the promotion effect of HG on *Bifidobacterium* and the inhibition effect of HG on *Escherichia-Shigella* were not significant in older men. Moreover, the promoting effect of HG on *Lactobacillus* was only significant in the young population.

During fermentation, changes in fecal microbiota structure of different populations are associated with changes in bacterial metabolites. The degradation products of carbohydrates by intestinal microbiota are mainly SCFAs, including Ace, Pro, Isob, But, Isov and Pen (29, 30). In this study, compared with the Ctrl group, the levels of total SCFAs were significantly increased in the HG group (Figure 3A). SCFAs are mediators of metabolic interactions between intestinal microbiota and hosts (31), they can regulate the activation immune cell functions by activating G protein-coupled receptors and inhibiting histone deacetylase (32, 33), and also promote intestinal homeostasis and oral tolerance (34, 35). Among them, the HG medium significantly increased the levels of Ace and Pro (Figure 3B). Pro has been shown to alleviate multiple sclerosis through immune regulation mechanisms (36), or reduce hypertension and cardiovascular injury (37). And Ace and Pro are important energy and signaling molecules (38). As other important metabolites for maintaining human health, the levels of Isob, But and Pen were no statistical difference between two groups, and were not significantly different between different populations (Figure 3). The reason may be due to the large individual differences, and more samples may be required to show significant differences.

The levels of each SCFAs were significantly correlated with various microbiota (Figure 5). Among the top 15 microbial groups by relative abundance, Ace, Pro, and But were significantly positively correlated with bacteria such as *Faecalibacterium* (39, 40), *Blautia* (41, 42), and *Megasphaera* (43, 44), however, they were significantly negatively correlated with *Escherichia-Shigella*. After fermentation, the relative abundance of *Faecalibacterium, Blautia* and *Megasphaera* in the HG medium was increased while that of *Escherichia-Shigella* was decreased. Therefore, it could be concluded that the levels of Ace, Pro, and But in HG medium were increased.

Carbohydrates that are available to intestinal microbiota can influence microbiota composition and the levels of gas and SCFAs produced (45). In addition, there are also significant differences in the abundance of intestinal microbiota among individuals in different regions, genders, and ages (46). In this study, we found gender- and age-associated variations in fecal microbiota. Fecal microbiota structures of the four populations showed significant differences after fermentation. During fermentation, fecal microbiota from women groups produced relatively more But, Pen, Isob, and Isov. The older groups produced relatively more But, while the young groups produced relatively more Pen. Apart from CH_4_, which was less affected by age and gender, the concentrations of the other four gases exhibited age- and gender-associated variations. For instance, the concentrations of CO_2_ produced by fecal microbiota of the older women population were relatively higher than those of the other groups. In contrast, the young population produced relatively more H_2_S, compared to the older population. While the women population produced relatively more H_2_S and NH_3_, relative to the men population. Therefore, people of different genders and ages had variations in fecal microbiota structures, which were further exacerbated by fermentation. These variations led to differences in microbiota products (such as SCFAs and gases) between the four populations.

In addition to SCFAs, gas production is an important index for maintaining intestinal microbiota balance (47). The composition and volume of intestinal gas are often adjusted by regulating the carbohydrates, proteins and fats in diets, which may achieve the purpose of preventing and treating the related gastrointestinal diseases (48). In this study, after 24 h of fermentation, fecal microbiota produced large amounts of CO_2_, H_2_ and small amounts of H_2_S, CH_4_, and NH_3_. Compared to the Ctrl group, the concentrations of CO_2_ and H_2_ in the HG group were significantly increased. As the final product of carbohydrate fermentation, H_2_ is a core component of metabolic homeostasis in the human gastrointestinal tract (49). H_2_S and NH_3_ are by-products of colonic bacterial fermentation of amino acids, such as methionine, cystine, cysteine, and taurine, which are detrimental to intestinal health (50). Both H_2_S and NH_3_ are linked to gastrointestinal diseases, such as ulcerative colitis, Crohn's disease and irritable bowel syndrome (51). The concentrations of NH_3_ were significantly reduced during the fermentation of the HG substrate, and the concentration of H_2_S was relatively reduced, indicating that HG is beneficial for intestinal health.

Intestinal microbiota composition may also affect the metabolic capacity of intestinal bacteria (52). CO_2_ is a key molecule in many biological processes (53). We found that CO_2_ was significantly negatively correlated with conditionally pathogenic *Escherichia-Shigella*, and significantly positively correlated with *Faecalibacterium*. And *Faecalibacterium* are potential indicators of gastrointestinal health (54). Therefore, the concentrations of CO_2_ in the HG group imply beneficial effects of the probiotics on human health. Similarly, there were significant correlations between *in vivo* H_2_ production and specific taxa of microbes, including *Bacteroides* and *Parasutterella*. Biologically, H_2_ has beneficial effects on the gastrointestinal tract (55). We found that H_2_ was significantly positively correlated with *Streptococcus* and negatively correlated with *Lactobacillus*. After HG intervention, the abundance of *Streptococcus* was highly increased, relative to that of *Lactobacillus*. Therefore, the concentration of H_2_ in the HG medium was increased. In addition, NH_3_ and H_2_S were significantly negatively correlated with *Bifidobacterium*, which were highly abundant. This may be the reason for the decreased concentration of NH_3_ and H_2_S in the HG medium.

At present, the utility of PICRUSt2 in predicting microbiota function has been validated (56). The results of KEGG showed that, among the top 15 metabolism-related functions in abundance, the addition of HG significantly increased the relative abundance of glycolysis/gluconeogenesis, biosynthesis of amino acids and secondary metabolites, amino acid (such as glycine, serine, threonine, cysteine, methionine) and nucleotide metabolism. At the same time, HG significantly suppressed pyruvate metabolism and oxidative phosphorylation, which eventually led to significant reduction in energy metabolism in the HG group. These results indicated that addition of HG change the metabolism pathway of fecal microbiota. Additionally, HG could significantly promote or inhibit certain metabolic functions in some populations. For example, the relative abundance of Biosynthesis of secondary metabolites increases significantly only in women populations. However, these metabolic functions did not differ significantly between the four populations (Supplementary Figures 3, 4).

In general, during fermentation, HG regulates the composition of fecal microbiota by inhibiting the growth of *Escherichia-Shigella* and *Bacteroides* while promoting the abundance of *Bifidobacterium, Lactobacillus*, and other beneficial bacteria, so as to regulate the metabolic products of fecal microbiota, including SCFAs and gases. Moreover, different groups may be regulated by HG due to differences in fecal microbiota and present different characteristics. However, the metabolic mechanism of HG needs to be further studied.

## Conclusions

Our study identified that the fermentation of HG changed the fecal microbial composition by increasing the abundance of beneficial bacteria such as *Bifidobacterium, Faecalibacterium, Prevotella* and reducing the abundance of harmful bacteria such as *Escherichia-Shigella*. As important metabolites of the fecal microbiota, the total production of SCFAs and gases was significantly increased in the HG group, among which the production of Ace, Pro, CO_2_, and H_2_ was significantly increased, while the production of Isov and NH_3_ was significantly decreased. Additionally, the supplement of HG showed certain differences in the regulation of microbiota from four populations. HG significantly increased the relative abundance of *Bifidobacterium* and significantly decreased the relative abundance of *Escherichia-Shigella* in the populations other than older men. The relative abundance of *Lactobacillus* was significantly increased in young populations. And the relative abundance of *Bacteroides* was significantly decreased only in the young women. Moreover, HG significantly increased H_2_ concentration in older men group. Taken together, HG could regulate the structure of fecal microbiota and its metabolites in a better direction, but different populations may be regulated by HG due to differences in fecal microbiota and present different characteristics. This should be taken into account when HG participates in precision nutrition formulations as functional foods.

## Data Availability Statement

The datasets presented in this study can be found in online repositories. The names of the repository/repositories and accession number(s) can be found at: https://www.ncbi.nlm.nih.gov/, PRJNA774123.

## Ethics Statement

The studies involving human participants were reviewed and approved by the Ethics Committee of the Hangzhou Center for Disease Control and Prevention (No. 202047). The patients/participants provided their written informed consent to participate in this study.

## Author Contributions

XP: funding acquisition, data curation, formal analysis, and writing—original draft. ZY: data curation, formal analysis, and methodology. XY: data curation and formal analysis. ZD: sample collection. WL: methodology, supervision, project administration, and review. All authors contributed to the article and approved the submitted version.

## Funding

This work was supported by Hangzhou Agricultural and Society Development Project (Grant No. 202004A20).

## Conflict of Interest

The authors declare that the research was conducted in the absence of any commercial or financial relationships that could be construed as a potential conflict of interest.

## Publisher's Note

All claims expressed in this article are solely those of the authors and do not necessarily represent those of their affiliated organizations, or those of the publisher, the editors and the reviewers. Any product that may be evaluated in this article, or claim that may be made by its manufacturer, is not guaranteed or endorsed by the publisher.
