# Supplementary material for: Effects of Zymosan on Short-Chain Fatty Acid and Gas Production in in vitro Fermentation Models of the Human Intestinal Microbiota
Source: Front Nutr. 2022 Jul 4;9:921137. doi: 10.3389/fnut.2022.921137 (PMC9291218; doi:10.3389/fnut.2022.921137)
Supplement: Supplementary file 1 [file Image_1.pdf]

Supplementary Material

1 Supplementary Figures

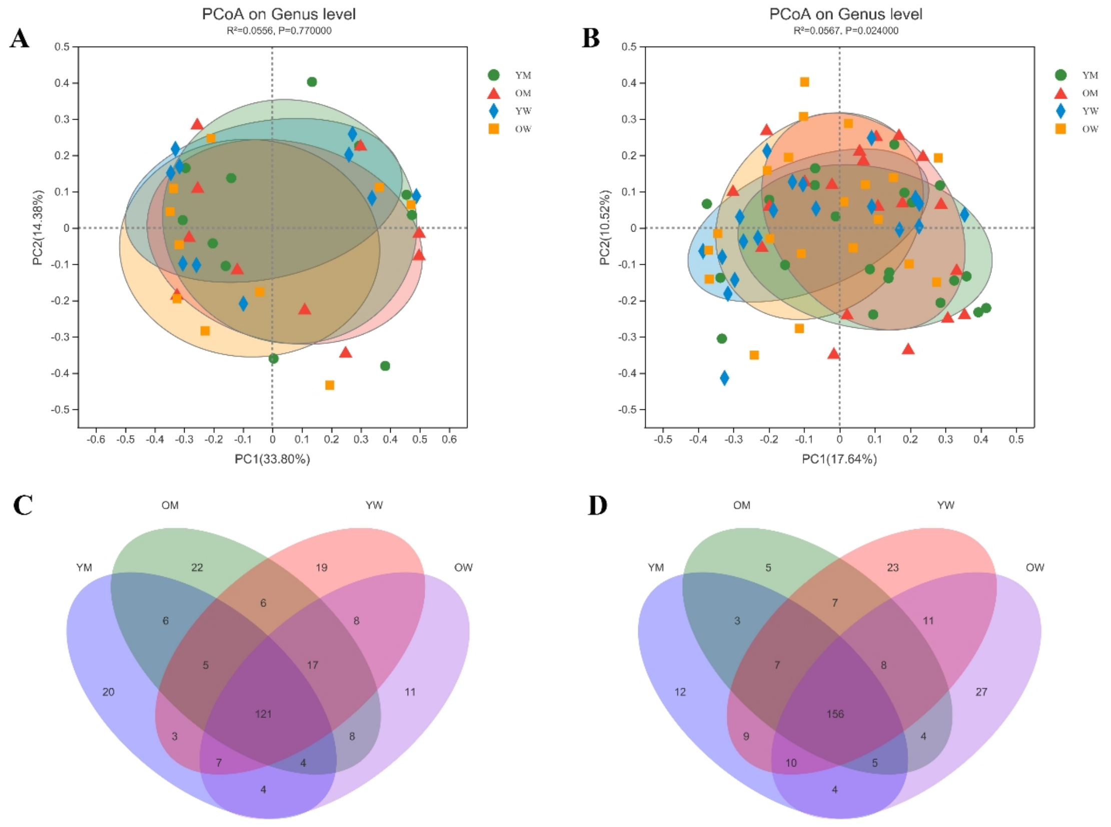

Supplementary Figure 1. Difference analysis of bacterial microbiota before and after fermentation. PCoA diagram of fecal microbiota at the genus level of the four populations before (A) and after (B) fermentation; Veen diagram of the fecal microbiota at the genus level of the four populations before (C) and after (D) fermentation.

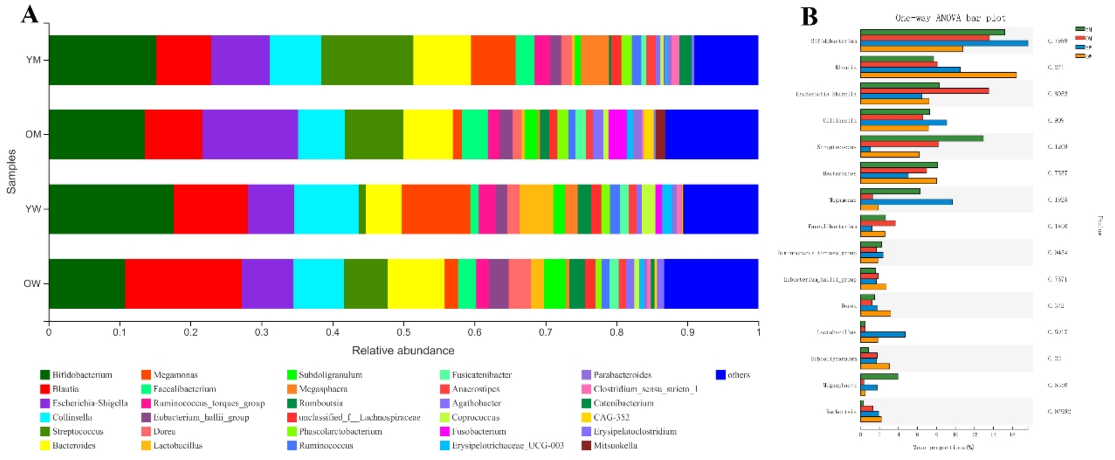

Supplementary Figure 2. Compositional (A) and multigroup comparative analysis (B) of the original fecal microbiota at the genus level of the four populations. 41 independent experiments × 3 replication experiments.

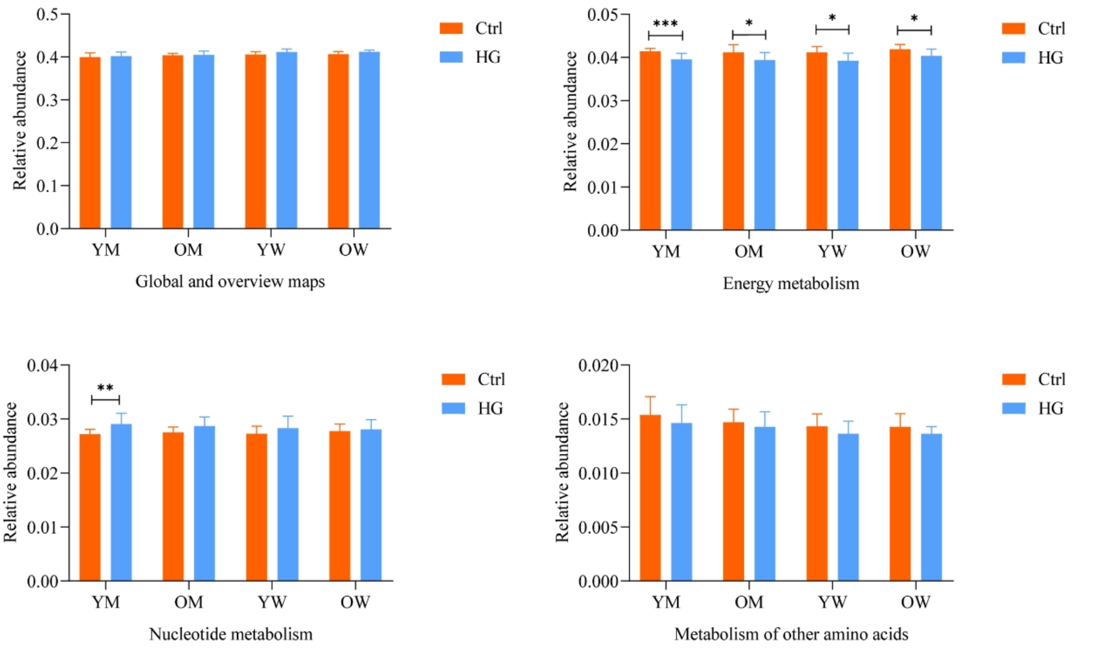

Supplementary Figure 3. Metabolic pathway abundance (level 2) of KEGG categories in the four populations. Data are means ± SD (41 independent experiments × 3 replication experiments). Statistical significance thresholds were: *, $0.01<p\leq0.05$; **, $0.001<p\leq0.01$; ***, $p\leq0.001$.

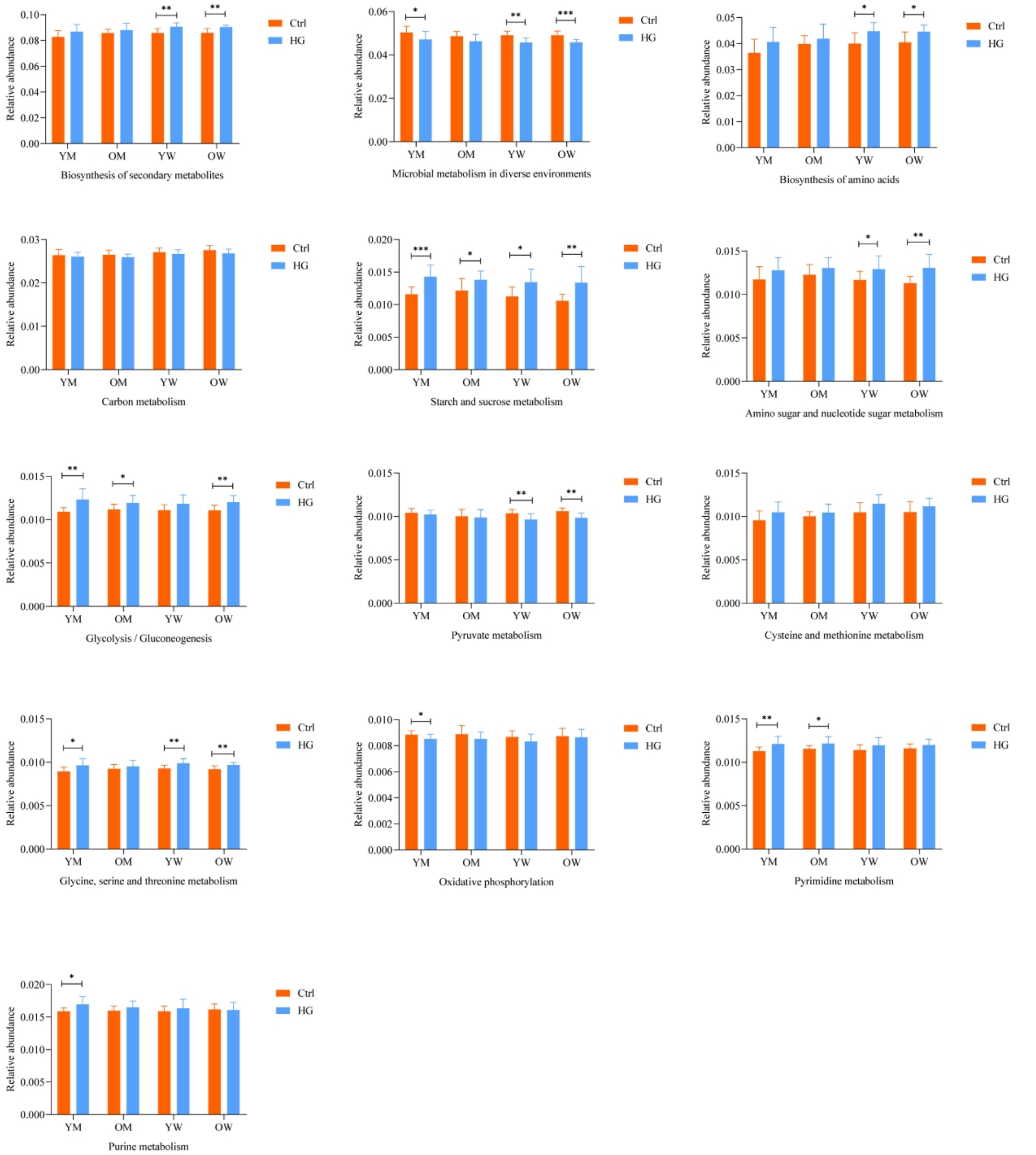

Supplementary Figure 4. Metabolic pathway abundance (level 3) of KEGG categories in the four populations. Data are means ± SD (41 independent experiments × 3 replication experiments). Statistical significance thresholds were: *, $0.01<p\leq0.05$; **, $0.001<p\leq0.01$; ***, $p\leq0.001$.
